# Experimental comparisons of passive and powered ankle-foot orthoses in individuals with limb reconstruction

**DOI:** 10.1186/s12984-018-0455-y

**Published:** 2018-11-21

**Authors:** Elizabeth Russell Esposito, Kelly A. Schmidtbauer, Jason M. Wilken

**Affiliations:** 1 Center for the Intrepid, Department of Rehabilitation Medicine, Brooke Army Medical Center, JBSA Ft, Sam Houston, TX USA; 2 Extremity Trauma and Amputation Center of Excellence, JBSA Ft, Sam Houston, TX USA; 3 Center for Limb Loss and Mobility, VA Puget Sound Health Care System, Seattle, WA USA; 4 University of Iowa, Iowa City, IA USA; 5 Department of Rehabilitation Medicine, Uniformed Services University, Bethesda, MD USA

**Keywords:** Powered exoskeleton, Mechanical work, Gait, Biomechanics, Metabolic cost, Limb salvage

## Abstract

**Background:**

Ankle-foot orthoses (AFO) are commonly prescribed to provide functional assistance for patients with lower limb injuries or weakness. Their passive mechanical elements can provide some energy return to improve walking ability, but cannot restore plantar flexor push-off. Powered AFOs provide an assistive torque about the ankle to address the limitations of passive devices, but current designs have yet to be implemented on a large scale clinically. Purpose: To compare passive AFOs to a new untethered, powered AFO design in a clinical population with lower limb reconstruction.

**Methods:**

A crossover study design, conducted on three individuals with lower limb reconstruction, compared gait mechanics at a standardized speed (based on leg length) in 4 AFO conditions: 1. None (shoes only), 2. Blue Rocker (BR, Allard, USA), 3. Intrepid Dynamic Exoskeletal Orthosis (IDEO), and 4. PowerFoot Orthosis (PFO BionX Medical Technologies, Inc.). The PFO was a custom, battery-powered device whose damping and power were capable to being tuned to meet patient needs. Subjects performed biomechanical gait analysis and metabolic testing at slow, moderate and fast speeds. Dependent variables included total limb power (calculated using a unified deformable segment model), mechanical work, mechanical efficiency, ankle motion, net metabolic cost across three speeds, and performance measures were calculated. Effect sizes (d) were calculated and d > 0.80 denoted a large effect.

**Results:**

Net positive work (*d* > 1.17) and efficiency (*d* > 1.43) were greatest in the PFO. There were large effects for between limb differences in positive work for all conditions except the PFO (*d* = 0.75). The PFO normalized efficiency between the affected and unaffected limbs (*d* = 0.50), whereas efficiency was less on the affected limb for all other conditions (d > 1.69). Metabolic rate was not consistently lowest in any one AFO condition across speeds. Despite some positive results of the PFO, patient preferred their daily use AFO (2 IDEO, 1 BR). All participants indicated that mass and size were concerns with using the PFO.

**Conclusions:**

A novel PFO resulted in more biomimetic mechanical work and efficiency than commercially-available and custom passive AFO models. Although the powered AFO provided some biomechanical benefits, further improvements are warranted to improve patient satisfaction.

## Introduction

Severe lower extremity injury often leads to musculoskeletal weakness and functional deficits [1]. Lower limb muscles (including the plantar flexors) are often affected, impairing the limbs ability to provide body support, forward propulsion, swing initiation, balance control, and foot clearance during swing [2–5]. Injuries to the calf musculature are particularly devastating due to the critical importance of the ankle in providing support for body position, and in propelling the body forward economically during common functions such as level-ground walking and the ascent and descent of stairs and slopes [6].

Ankle-foot orthoses (AFO) are commonly prescribed to provide functional assistance for patients with lower limb injuries or weakness. Passive AFOs have been shown to be effective at improving gait and performance in patients with musculoskeletal weakness [7]. They rely on passive mechanical elements such as springs, dampers, or flexible struts to improve walking ability. Muscles surrounding the ankle joint in uninjured individuals generate more positive mechanical work than other muscle groups in the body [8]. However, because of the passive spring like nature of traditional AFOs, they only return to a neutral position when unloaded, rather than producing the peak power and work observed in the intact ankle. As a result, gait quality and performance may still be limited [9–13].

There have been numerous important advancements in the design of passive ankle assistive devices over recent years [14]. However, in response to the limitations of passive AFOs, powered AFOs were first introduced into the literature in 2005 as a proof-of-concept [15] and numerous models have been developed over the past 12 years [16–21]. Generally, a powered AFO addresses the inherent limitations of passive devices by providing a tunable assistive torque about the ankle joint [22–24]. They facilitate greater range of motion, active damping upon heel strike, and powered push-off, but they are often heavier and bulkier than passive designs, which can be energetically unfavorable. In addition, the power requirements have often led to these devices being tethered to external power and electronics [15, 23]. Although their use has primarily been limited to a laboratory setting, more recent efforts have incorporated untethered, autonomous designs as the next progression in powered AFO development [24–27].

Advances in untethered designs are an important step towards the clinical incorporation of these devices. The vast majority of powered AFOs and exoskeletons have been tested on able-bodied individuals [15, 19, 27–33], and there have been ongoing efforts to introduce these designs clinically [17, 20, 34–36], but more research is still needed to better understand how different clinical populations interact with these devices [21]. For example, various designs have been tested in stroke patients, in whom plantar flexor weakness is common [36], but there are additional opportunities to explore the efficacy of these designs in patients with lower limb reconstruction whose weakness stems from traumatic injury. Powered prosthetic ankles have been commercially available for years (e.g. the BiOM by BionX Medical), but powered AFOs have yet to make this crucial leap, despite the large numbers of patients who undergo limb reconstruction surgeries [37, 38], often in lieu of amputation. There are inherent challenges to working around a biological limb with musculoskeletal deficiencies, but if a powered AFO can assist ankle joint function without additional burden to the energy costs of the user, it may be a useful clinical tool for rehabilitation. In this study, we aimed to introduce a new powered AFO design to a clinical patient population of individuals with lower limb reconstruction who had plantar flexor weakness. Specifically, this study incorporated a comparative effectiveness of currently available passive designs to the new powered design. We hypothesized that the powered AFO would offer performance improvements in metabolic energy expenditure, mechanical energy generation, and in a battery of performance measures.

## Methods

### Ankle-foot orthoses

**Fig. 1:**
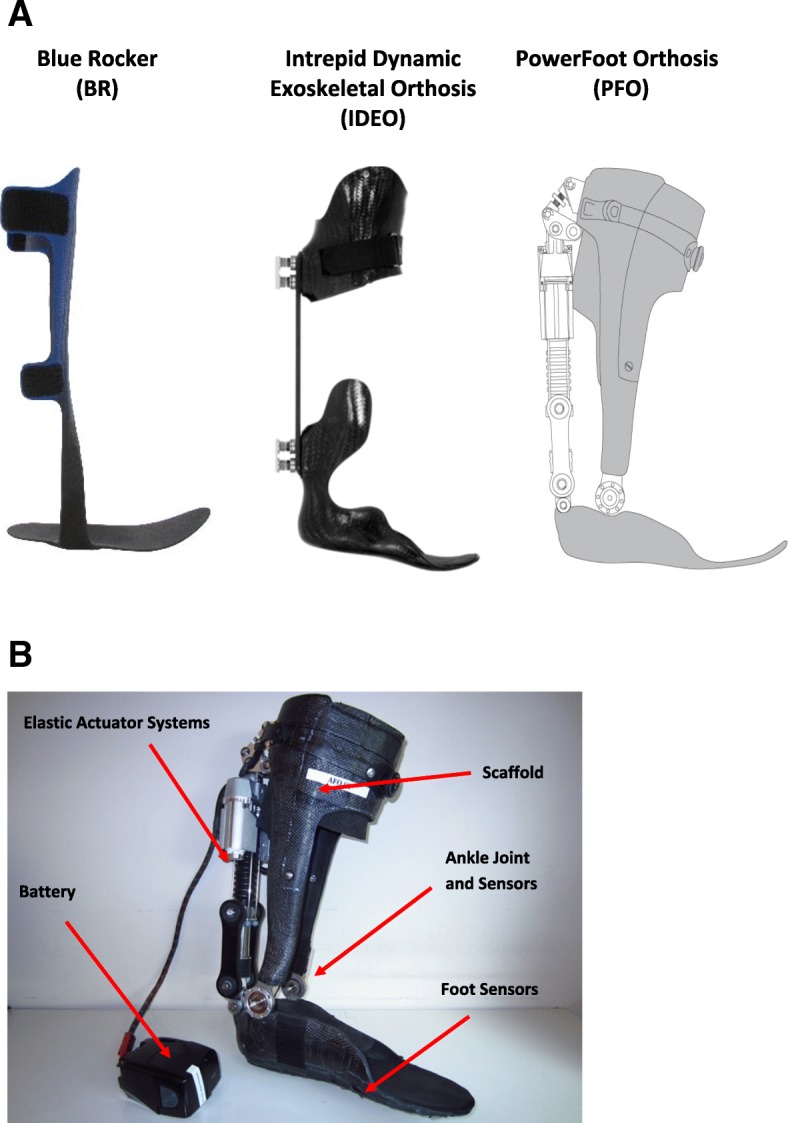
**a** Study devices worn by each individual. From left to right, Blue Rocker (BR; Allard, USA), Intrepid Dynamic Exoskeletal Orthosis (IDEO), and schematic of the PowerFoot Orthosis (PFO; BionX Medical Technologies Inc., USA). **b** Rear view of the PFO and componentry

A repeated measures study design was used to compare biomechanical, metabolic, and performance-based metrics in the four conditions described below and in Fig. 1a).

- **Blue Rocker** (BR condition, Allard, USA): The BR is commercially available carbon fiber AFO with cuff below the knee, lateral strut, and flexible foot plate.
- **Intrepid Dynamic Exoskeletal Orthosis** (IDEO): The IDEO is a custom carbon fiber AFO with cuff below the knee, lateral strut and rigid foot plate that has been described previously [39, 40].
- **None**: The None condition refers to walking with shoes only and without an AFO.
- **PowerFoot Orthosis** (PFO, BionX Medical Technologies, Inc., USA).

The AFOs included were intended to represent three categories of devices: BR – passive conventional AFO, IDEO – passive dynamic advanced AFO, PFO – powered advanced AFO. The PFO is a computer-controlled ankle-foot orthosis in which joint position, impedance, and torque are varied in response to walking phase and step-to-step gait variations. The PFO is comprised of a series-elastic actuator [41], motor controller, a state controller, and a scaffold structure (Fig. 1 b). At the ankle joint, angular sensors measure angle and angular velocity. Calibrated spring systems calculate torque at this joint. The state controller uses inertial measurement units to compute walking speed and shank trajectory. These sensors are also used to detect early and late stance, as well as swing phases. The state controller integrates the sensing information, makes decisions for walking, and sends command to the motor controller, which controls the series elastic actuator. The actuator system is integrated on the scaffold structure. Power electronic components in actuator systems are placed next to the motor, and the Lithium-Polymer battery is connected to the orthosis via a cable and, to reduce distal mass, is stored in a pack worn about the waist. The actuator is controlled to generate impedance during initial foot contact and then net positive work at the orthotic ankle joint for powered orthotic plantar flexion during push-off. After toe-off, assistance is provided as needed to raise the foot into a more dorsiflexed position to allow toe-clearance and prepare for foot strike. The scaffold is customized for each patient by a certified orthotist and consisted of a carbon fiber shank and footplate, affixed with a hinge near the ankle joint.

A wireless communication system (Bluetooth) allows ankle stiffness and power delivery to be adjusted in real time while the user walks with the orthosis. Device position, impedance, and torque control can be customized on an individual basis. The magnitude and timing of power delivery are measured directly from sensors within the orthosis and then initially adjusted for each wearer to match the performance of a biological ankle. Additional tuning is based on patient feedback. The device is also designed to generate varying power assistance according to walking speed and terrain (ramps and stairs). The control configuration is autonomous. The PFO is designed for users up to 113 kg body mass, and it has an orthotic ankle joint motion range of 15° dorsiflexion and 25°plantar flexion.

Average AFO device masses were as follows: BR: 0.3 kg, IDEO: 1.9 kg IDEO, PFO: 3.4 kg.

### Subjects

Three subjects participated in this case series (Table 1). Subjects were male Service Members who had sustained lower extremity injury requiring surgical management and continued AFO use for walking and running. P01 and P02 habitually wore the IDEO as their daily device for all activities and P03 wore the Blue Rocker for walking and the IDEO for running.

**Table 1:** Subject characteristics. Average ankle plantar flexor power deficits were relative to the sound limb during walking. Subjects wore the same footwear during all testing conditions

Subject	Age (years)	Height (m)	Mass (kg)	Limitations in ankle strength or ROM	% Ankle power deficit	Footwear
P01	38	1.93	76.2	PF weakness DF weakness	30%	New Balance 990
P02	23	1.97	100.4	PF weakness	23%	New Balance 910 VI
P03	24	1.74	90.9	PF weakness 25° ankle ROM	60%	Apex Rhino Runner

### Equipment

A 26-camera motion capture system (120 Hz; Motion Analysis Corp, Santa Rosa, CA) with five centrally-located force platforms (1200 Hz; AMTI, Watertown, MA) captured data along a 20 m walkway. A detailed description of marker placements and the full body model has been previously described [42]. Oxygen consumption was recorded using indirect calorimetry and a portable, telemetered metabolic unit (K4b2, Cosmed, Inc., Rome, Italy) [43]. A heart rate (HR) monitor (Polar Electro Inc., Lake Success, New York) was worn about the chest.

### Protocol

The extent of conditions and metrics tested required that data be collected with each AFO on a separate day. The order of testing was randomized across AFO conditions. Marker trajectory and force data were collected as subjects walked through the capture volume at a self-selected speed and at a standardized speed based on leg length using a Froude number of 0.16. A consistent controlled speed was used to allow equivalent task demands across subjects (e.g. those with shorter legs walked slower than those with longer legs). During metabolic data collection, subjects rested in a seated position for a minimum of 10 min, or until baseline metabolic rate was achieved, with no change in average rate over a minute period. Patients then participated in metabolic testing at three speeds, all of which were scaled to leg length using Froude numbers of 0.10, 0.16, and 0.23. Subjects walked until steady state metabolic rate was achieved for at least two minutes, as confirmed from visual inspection of the data. Agility and mobility were assessed by having participants complete two trials of the T-test and four trials of the 4-square step test in each AFO condition [44]. Briefly, the T-test is designed to test speed and agility and involves forward and backwards runs, as well as side shuffles. The 4-square step test is designed to test agility and weight transfer and involves rapid stepping forwards, backwards, and sideways over small pipes.

Device preference was ranked at the final testing session, and subjects were encouraged to provide open-ended feedback to support their preference rankings.

### Analysis

Biomechanical data were analyzed from the standardized speed and the self-selected speed for each AFO condition is included as a descriptive measure. Kinematic data were interpolated and filtered using a 4th order low-pass Butterworth filter with a cutoff frequency of 6 Hz; kinetic data were filtered with a cutoff frequency of 50 Hz. Peak ankle angles, ranges of motion, and internal joint moments were calculated. Power was calculated using a unified deformable (UD) segment model to quantify power from all structures below the knee [34]. UD power was integrated over time to calculate mechanical work (J/kg), and a dimensionless efficiency metric was then calculated as the ratio of positive to negative work. All kinetic data were scaled to total system mass (biological body mass plus AFO mass).

Baseline metabolic rate was calculated as the average metabolic rate during the final 2 min of seated rest, and the values were subtracted from walking values. Net steady state metabolic rate during walking was calculated as the average metabolic rate during the final 2 min of walking. All metabolic data were scaled to biological body mass, exclusive of orthosis mass. Thus, metabolic rate was calculated in units of mL O_2_ per kilogram biological body mass per minute.

The fastest time for the 4-square step test and T-test were recorded and used for analysis. Trials in which the subject touched the testing equipment during the 4-square step test were discarded and not repeated.

The outcome measures assessed were 1) Preference, 2) Ankle range of motion, 3) Bilateral UD power, 4) Bilateral mechanical work (positive and negative), 5) Bilateral efficiency, 6) Net metabolic cost, 7) Fastest t-test time, 8) Fastest 4-square step test time. Biomechanical data were first averaged across trials and then averaged across subjects. As this was a case series, a full statistical analysis was not performed. Instead, descriptive statistics (mean values, standard deviations) were calculated across subjects and percent increases/decreases compared between AFO conditions. For data collected bilaterally, effect sizes were calculated to compare between limbs in the same condition with the assumption that the unaffected limb served as a control. Effect sizes greater than 0.2, 0.5 and 0.8 indicated small, moderate, and large effects, respectively, between limbs [45].

## Results

### Preference

Subjects preferred the AFO(s) they wore on a daily basis (Table 2). The additional mass was the primary criticism of the PFO, and all subjects noted their preference for the PFO would increase if the device were lighter.

**Table 2:** Self-selected walking speed, and time to complete the T-test and 4-Square Step Test. Participants who experienced difficulty or inability to complete a test are indicated

Subject Ranked Preference	1st	2nd	3rd	4th
P01	IDEO	PFO	NONE	BR
P02	IDEO	PFO	BR	NONE
P03	BR	IDEO	PFO	NONE

P01 rated the PFO second. He appreciated the ability of the PFO to automatically change the amount of power with speed and terrain, but preferred the IDEO because it offered the most mobility with the least amount of pain, was not limited by battery life, and did not cause pain in lateral movements. The BR was rated last due to pain during lateral movements. P01 frequently lost power during testing sessions and, as a result, trials were repeated several times.

P02 rated the PFO second. He reported that the PFO felt like walking pre-injury, which he had not experienced with any other AFO device. The PFO was not his preferred device overall but he did prefer it to the other AFOs for over ground and inclined walking, and on stairs. However, P02 found the performance tasks more difficult in the PFO. He indicated the backwards running portion of the T-test was more difficult with the PFO because it did not supply the necessary power he wanted/needed and it was heavy.

P03 rated the PFO third. He disliked the bulk and mass of the PFO and experienced difficulties donning the device. Difficulty with lateral movements also contributed to the PFO being rated third. However, he did report that the PFO facilitated an increased walking speed and modulated stiffness, reporting that it felt like he was “walking on pavement instead of sand.”

### Range of motion

**Fig. 2:**
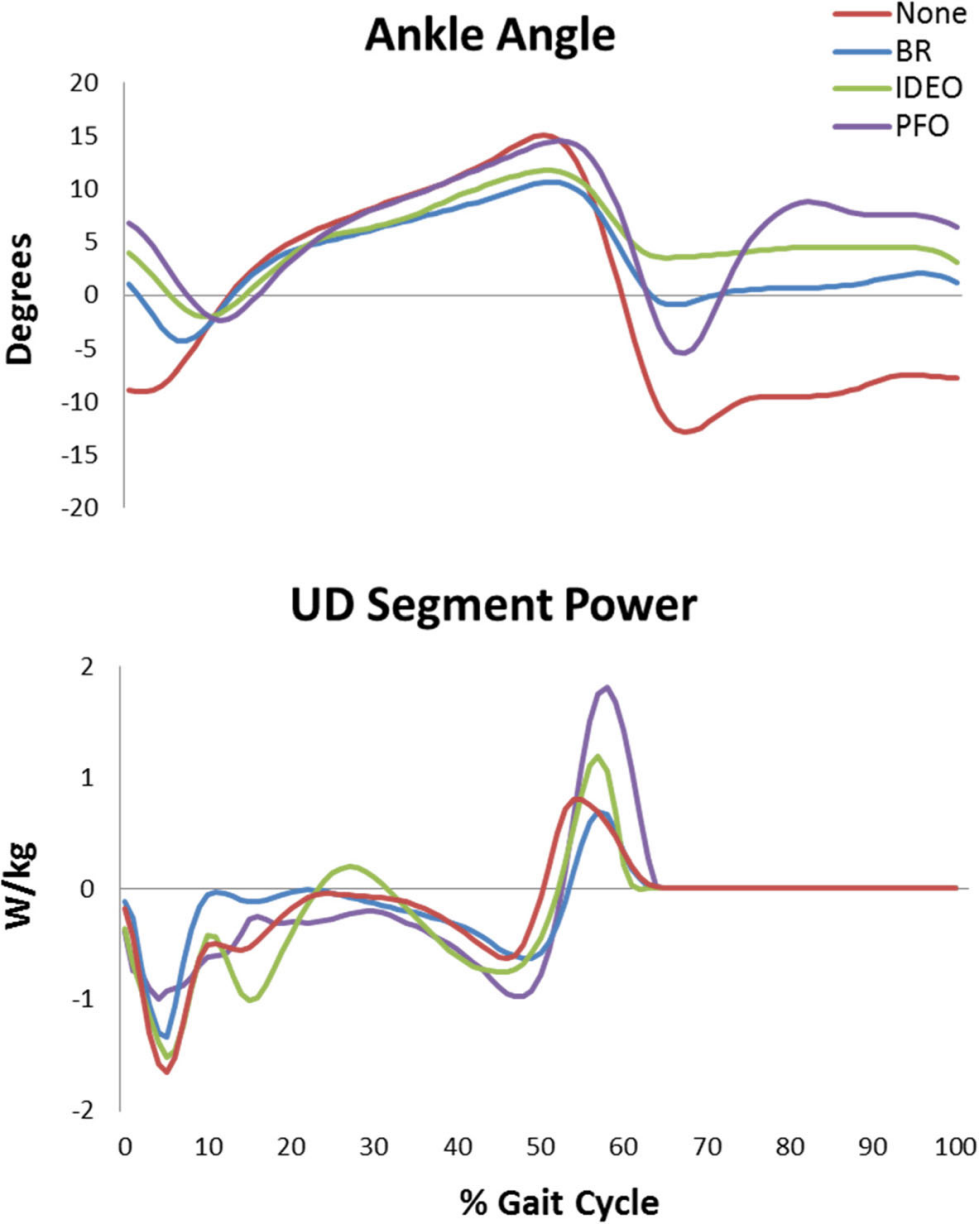
Mean ankle angles (dorsiflexion +/plantar flexion -) and unified-deformable (UD) segment power of the affected limb across the gait cycle in each orthosis condition. Data were averaged across trials then across subjects. Kinetic data were scaled to total system mass, inclusive of orthosis mass

The PFO provided greater ankle range of motion than the semi-rigid passive devices (IDEO and BR) by providing plantar flexion at push-off combined with greater dorsiflexion during swing (Fig. 2). Both semi-rigid passive AFOs restricted ankle ROM compared to not wearing a device.

### Power

The PFO increased peak UD power generation at push-off in the affected limb relative to all other conditions (Fig. 2). For example, compared to None condition, the PFO increased push-off power an average of 54, 50 and 105% for P01, P02, and P03, respectively. On the unaffected side, push-off power generation with the PFO was lower with large effect size for None (15.3% less, *d* = 2.70) and BR (7.5% less, *d* = 1.20), and a moderate effect for greater push-off power generation in the IDEO condition (5.2% more, *d* = 0.77).

### Mechanical work

**Fig. 3:**
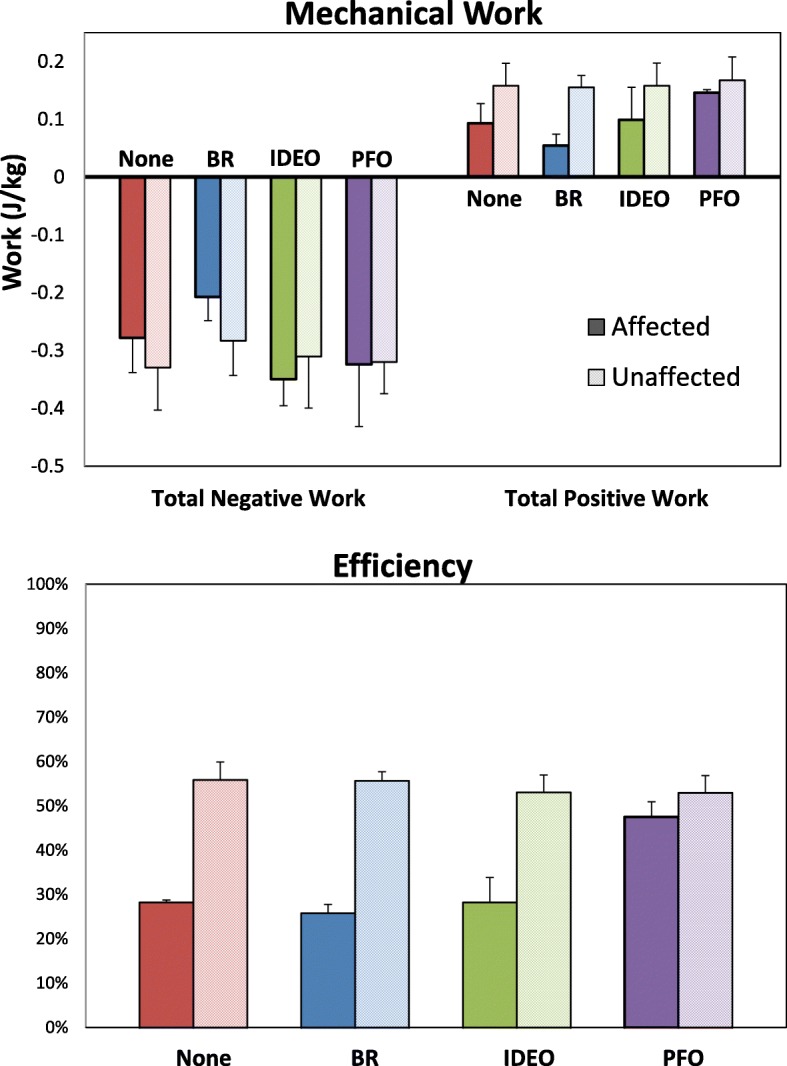
Mean (error bars are 1SD) negative and positive mechanical work, and mechanical efficiency of the affected (solid bars) and unaffected (shaded bars) limbs in each orthosis condition. All kinetic data were scaled to total system mass, inclusive of orthosis mass

The unaffected limb generated more positive mechanical work than the affected limb in the None (*d* = 2.35), BR (*d* = 5.02), and IDEO (*d* = 1.22) conditions (Fig. 3). There was only a moderate effect for greater net positive mechanical work on the unaffected side in the PFO (*d* = 0.75). When comparing across conditions on the affected limb, the PFO consistently generated the greatest net positive mechanical work (*d* > 1.17), and the BR generated the lowest (*d* > 1.06).

Net negative work was only different between limbs in the BR condition (*d* = 1.47). Effect sizes ranged from no effect in the PFO (*d* = 0.05) to moderate effects in the IDEO (*d* = 0.55) and None (*d* = 0.76) conditions. There were differences across conditions on the affected limb. The BR consistently had the lowest net negative mechanical work (*d* > 1.37), and there was a large effect for greater net negative mechanical work in the IDEO compared to the None condition (*d* = 1.33). There were only small-moderate effects for differences between the PFO and passive AFO conditions (IDEO: *d* = 0.31, BR: *d* = 0.53).

### Efficiency

When using the unaffected limb as a control, there were large effects between limbs in mechanical efficiency for the None condition (*d* = 3.701, BR (*d* = 6.69), and IDEO (*d* = 1.69) (Fig. 3). There was only a small effect (*d* = 0.50) for differences between limbs in the PFO condition, indicating that it may have restored mechanical efficiency to a more normative level. When comparing across conditions on the affected limb, the PFO had greater mechanical efficiency than the BR (*d* = 2.40), IDEO (*d* = 1.39) and None (*d* = 1.40) conditions. The BR had the lowest mechanical efficiency values and there was a large effect for differences between the BR and None conditions (*d* = 1.05).

### Net metabolic cost

**Fig. 4:**
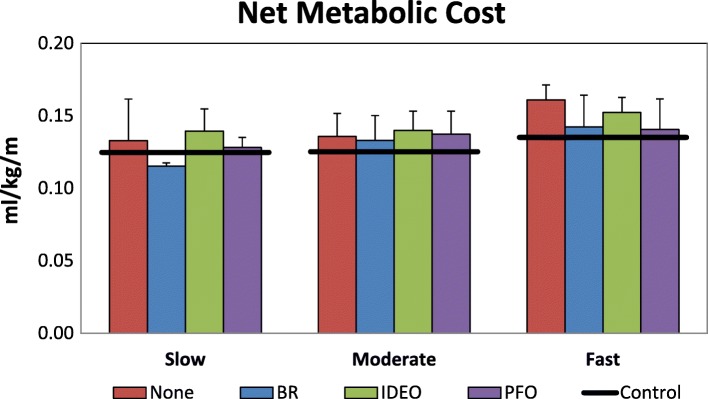
Mean (error bars are 1 SD) net metabolic cost at a slow, moderate, and fast speed in each orthosis condition. All metabolic data were scaled to biological body mass, exclusive of orthosis mass. Black lines indicate average control data for able bodied individuals at each speed to serve as a reference. Reference data are adapted from Russell Esposito et al., JRRD, 2014 [55]

No single device consistently provided the lowest net metabolic cost across speeds (Fig. 4). At the fastest speed, when ankle plantar flexor power could contribute the most to forward propulsion, the PFO had the lowest average metabolic rate. At slower speeds, the tradeoff between the consequence of added mass and the benefit of added power may have contributed to similarities between the PFO and No AFO conditions. Large effects between AFO conditions were found only at the slow and fast speeds, but not at the moderate speed. At the slow speed, the PFO had a lower net metabolic cost of walking than the IDEO, even despite its greater mass (*d* = 0.85). There were also large effects for a lower metabolic cost of walking in the BR compared to both the IDEO (*d* = 2.86) and PFO (*d* = 1.06). At the fastest speed, there was a large effect for a greater metabolic cost of walking in the None conditions compared to the PFO (*d* = 2.11) and None (*d* = 0.98) conditions.

### Performance measures

Self-selected walking speed, T-test times, and 4-square step test times were mixed across the four conditions with no noticeable trends across the three subjects (Table 3). Subjects did not walk the fastest in any single AFO condition, and P03 walked the fastest in the None condition. Two of the three subjects experienced PFO power losses during the T-test, which may have resulted in slower times than some of the other conditions. However, P03 experienced no power losses and had a substantially slower time in the PFO compared to the other conditions. None of the subjects experienced power losses during the 4-square step test. Again, P03 performed substantially lower in the PFO compared to the other conditions.

**Table 3:** Individual ranked preference of each orthosis condition (BR – Blue Rocker, IDEO – Intrepid Dynamic Exoskeletal Orthosis, PFO – PowerFoot Orthosis, None – no orthosis)

	BR	IDEO	None	PFO
Overground Self-Selected Walking Speed (m/s)				
P01	1.11	1.37	1.43	1.40
P02	1.25	1.50	1.24	1.39
P03	1.26	1.32	1.01	1.33
T-Test (sec)				
P01	Unable^a^	15.5	13.2	18.4^b^
P02	15.4	17.0	15.4	16.1^c^
P03	16.6	20.1	13.9	35.4
4-Square Step Test (sec)				
P01	4.2	4.2	3.5	3.9
P02	4.2	4.3	4.3	4.3
P03	4.3	5.1	4.7	7.2

^a^Unable to complete due to pain from the BR

^b^PFO power fluctuated throughout trial and pain level increased

^c^PFO lost power during the backwards run portion of the T-test

## Discussion

Powered exoskeletons have been useful for revealing important information about the musculoskeletal system during walking. Engineering efforts to replicate or restore function of the human lower leg have been effective when used to study isolated variables (e.g. the effect of powered push-off on the metabolic cost of walking [46]). Studies have even shown that augmenting ankle joint function with a powered assistive device can reduce the metabolic cost of walking below that of normal walking [23, 47, 48], but these studies have primarily been performed using uninjured, able-bodied individuals. Comparing to able-bodied individuals is useful for testing proof-of-concepts that are critical and necessary to advance device designs, but incorporating the intended user population is necessary for testing the applicability of the device. Few studies use a stand-alone untethered plantarflexion assist powered device in a patient population [17, 20, 49], but none have this state controller and actuator combination, and none evaluate individuals with traumatic injuries leading to limb reconstruction [21].

A powered device that is conceptually similar to the one tested here, with a spring and actuator in series, was used as part of a training intervention to improve gait following stroke [20]. Although the study results differ with respect to total motion, power, and other gait parameters, the substantial difference in study participants limits the relevance of direct comparison to this work. Another study used a similar control and actuator combination, and saw similar increases in dorsiflexion during swing, but the device was an exosuit, which is inherently a different device, since it also provides passive hip assistance, and again was applied to stroke patients [36].

Active powered devices, such as the PFO, are designed to replace or augment some amount of ankle function, thus allowing greater mobility both during and after rehabilitative interventions [41, 50]. However, there are considerable challenges when using powered AFOs in clinical populations. In this study we compared a powered AFO to passive designs to determine its efficacy in patients with lower limb reconstruction. Individuals with lower limb reconstruction often have musculoskeletal weakness and functional deficits requiring orthotic intervention. While passive orthoses can restore functional ability to a certain degree, they often restrict ROM and do not provide any supplementation to ankle push off [12]. The PFO provided larger ankle ROM and increased UD segment power generation during push off compared to the BR, IDEO and None conditions. The novel PFO restored a more biomimetic gait, specifically about the ankle joint, through the powered plantar flexion at push-off and dorsiflexion assist during swing. The PFO also produced greater positive mechanical work and greater efficiency than commercially-available and custom passive AFO models, as well as compared to not wearing an AFO. Of the two passive devices, the custom, carbon fiber IDEO provided greater positive mechanical work than the commercially-available BR. Although the powered AFO provided some biomechanical benefits, further design improvements are warranted to improve patient satisfaction and gait. Patients preferred the AFO(s) they used on a daily basis over the PFO. Mass and size were primary concerns with using the PFO, although all subjects understood that this was a prototype design.

Although the net metabolic cost of walking did not decrease when using the PFO, the PFO was able to reproduce net metabolic cost values similar to those when wearing much lighter passive AFOs (IDEO and BR). These results concur with other research that has shown that no significant decreases in the metabolic cost of walking are seen with healthy [46], elderly [51], or stroke patient [34] users wearing powered AFOs compared to a ‘None’ condition, though it should be noted that these were all tethered devices that weighed less than the untethered PFO in this study. Additional mass added to the body typically increases joint work and the metabolic cost of walking, particularly when added distally to the limbs [52]. The powered assistance from the PFO was able to offset this increase such that it approximated the lighter models. Therefore, if the mass of the PFO were reduced, it could be expected to reduce the metabolic cost of walking as well. This reduction may be particularly important at faster speeds, when the contributions of the ankle plantar flexors play a greater role [53].

### Limitations

This study was a preliminary test of a powered AFO design in individuals who had experienced lower limb injuries resulting in plantar flexor weakness. As such, the device was only worn within the laboratory environment and the overall accommodation time was relatively short. Subjects were required by protocol to wear it for at least 20 min prior to testing, during which time tuning parameters were optimized within a normative reference range based on patient feedback. However, all subjects had at least 2 h of wear time prior to any testing. It is unknown how accommodation time may have influenced the results of this study as accommodation time is an ongoing, but unresolved, discussion within the orthotics and prosthetics community [54]. In addition, this study utilized a heterogeneous patient population due to the inclusion of individuals who had experienced limb reconstruction. The participants also had modest impairments in function, as could be seen in data form the None condition. This was intentional due to the exploratory nature of this study and the desire to minimize risk should the device not function as planned. Lastly, the participants had high performance expectations due to their prior experience with high-performance carbon fiber bracing and high-energy activities. These patients received physical therapy that targeted sprinting, jumping, and maneuverability training. Although they were fully aware that this design was a prototype, their comments regarding device preference touched upon the limitations of the PFO for the high energy activities to which they are accustomed. It is unknown how the device may be rated if fewer or different activity conditions were tested as part of the study protocol.

## Conclusions

Considerable research has focused on developing assistive devices to improve function and reduce the metabolic cost of walking. A novel, untethered PFO used in a clinical patient population with lower limb reconstruction was effective at increasing range of motion, power, mechanical work, and efficiency relative to passive AFO designs, but it did not consistently reduce the metabolic cost of walking. The greater mass of the PFO relative to the passive designs may have strongly affected these results. Overall, patients preferred whichever AFO they used on a daily basis for walking but saw the potential future benefits of untethered, powered AFO designs.

## Abbreviations

AFO: Ankle Foot Orthosis
BR: Blue Rocker
HR: Heart Rate
IDEO: Intrepid Dynamic Exoskeletal Orthosis
PFO: Power Foot Orthosis
UD: Unified Deformable
